# Alveolar Abscess and Treatment of Dead Teeth

**Published:** 1885-12

**Authors:** H. H. Harrison


### Alveolar Abscess and Treatment of Dead Teeth.

BY DR. H. H. HARRISON.

I have been watching the practice of many operators in the dental profession for a considerable period, and it seems to me that there is, with very many, a lack of proper conception, or want of a true physical diagnosis in the pathological condition of Alveolar Abscess, and also a misinterpretation of the principles involved in the treatment of dead teeth.

It is a very important feature in any diseased condition to know its true character and the cause that produces it if possible, before we can intelligently treat the case. To blindly ignore this and ignorantly treat disease, seems to have at least the odor of quackery. In order to ascertain the true condition of any disease, we must begin with the most prominent symptom and follow every avenue in detail, recording at each successive step any fact that may be developed.

In the first place, abscess in any part of the body is the result of inflammation. And now, as we proceed, we must, if possible, discover the cause of the inflammation, and while this is obscure in many cases, in others it is perfectly plain, and such it is in Alveolar Abscess as a rule. We have inflammation of the nerve of a tooth, caused by exposure to foreign substances, by thermal changes or by accident, assisted possibly by an enfeebled or depraved condition of the general system. This, of course, produces periostial inflammation, but may not result in abscess. But a continued inflammation produces death of the nerve, as it does in any other tissue of the body. After death comes decomposition; nature in reducing the once living structure to its original elements, accomplishes its work by division and subdivision. In this chemical laboratory are manufactured poisonous gases inimical to vital tissue, which require more space than the living nerve, they find their exit at the most vulnerable point of this bony enclosure—namely, the apical foramen of the root, which but for this would yield to the extreme pressure. As they pass through the apex opening they come in contact with and poison the living tissue, producing inflammation of the surrounding parts. Nature, true to herself, throws around the diseased part, a cordon or sac to prevent further injury, and to carry away the wasting elements. This is the true alveolar abscess that gives us so much trouble and our patients so much pain.

I do not propose to offer any new treatment for this condition, but only wish to connect it with the treatment of dead teeth, to explain a principle not recognized by many in this operation.

Taking the foregoing as the correct theory of alveolar abscess, we advance to the treatment of dead teeth preparatory to filling.

If the decomposition of the animal tissue results in inflammation and abscess, then the removal of the dead nerve before abscess has been established, will prevent its formation. Following on logically we can confidently say, that the nerve cavity being thoroughly filled with an incorruptible and healthful material, we shall never have abscess in a tooth thus treated at any subsequent period, at least from the cause just mentioned.

In this preparatory treatment of dead roots we ought to rely more on the instrument and not so much on medicine for cleansing. Nerve broaches, drills and excavators are better disinfectants for this purpose than any medicine you will find, and in four-fifths of the cases presented you can fill at first sitting as well as treat with medicines for a month and then fill. Indeed I am almost ready to say that I cannot conceive a case in which success is dependent upon treatment with disinfectants, for if you cannot penetrate with an instrument, the filling operation is only an experiment, and could be made no more certain by a disinfecting treatment. 'Tis true it might be a benefit for a brief period by neutralizing the poisonous material within the cavity, but its effect would soon pass, and as soon as the cavity filled with fluid, subject to decomposition, trouble would arise.

Hence, I seldom use disinfectants, but fill as soon as cleansed with the instruments, relieving my patients of frequent sittings, and thereby keeping my business under better command. A short time since saw a case where a lateral incisor had been treated with disinfectants for two years, the tooth quite black, and no attempt had been made to fill at all, and I don't know when it is to be filled—possibly not until the millenium sets in. This by a reputable operator in a city of our own State.

In conclusion, let me say a few words about the filling operation. Every operator chooses his own plan and selects his own material, and I care but little about this if he makes it a success, for I think success is not dependent alone upon one man's pet plan of operating. Of course some plans seem to be more practical than others, and we should all strive to be practical. But the principle involved is certainly to make a filling of incorruptible, healthful material, perfectly adapted to all the inequalities of the cavity made vacant by the removal of the nerve. This covers all the ground, and you can exercise your own individuality in accomplishing this end, and success will be yours and your patient will be happy.

